# SeedsGraph: an efficient assembler for next-generation sequencing data

**DOI:** 10.1186/1755-8794-8-S2-S13

**Published:** 2015-05-29

**Authors:** Chunyu Wang, Maozu Guo, Xiaoyan Liu, Yang Liu, Quan Zou

**Affiliations:** 1 School of Computer Science and Technology, Harbin Institute of Technology, No.92 West Dazhi Street, Nangang District, Harbin 150001, China; 2 Department of Computer Science, Xiamen University, No.422, Siming South Road, Xiamen 361005, China

## Abstract

DNA sequencing technology has been rapidly evolving, and produces a large number of short reads with a fast rising tendency. This has led to a resurgence of research in whole genome shotgun assembly algorithms. We start the assembly algorithm by clustering the short reads in a cloud computing framework, and the clustering process groups fragments according to their original consensus long-sequence similarity. We condense each group of reads to a chain of seeds, which is a kind of substring with reads aligned, and then build a graph accordingly. Finally, we analyze the graph to find Euler paths, and assemble the reads related in the paths into contigs, and then lay out contigs with mate-pair information for scaffolds. The result shows that our algorithm is efficient and feasible for a large set of reads such as in next-generation sequencing technology.

## Introduction

The introduction of the massively parallel next-generation sequencing (NGS) technologies has caused a great increase in the number of reads typically generated by experiments. At the same time, the shorter read length from NGS and the sheer demand for more scalable assemblers have been an important computational challenge, and the genome assembly continues to represent one of the most difficult and important algorithmic problems in bioinformatics. Software technology and algorithm implementation become critical factors when dealing with terabytes of data. Cloud computing as a brand new way of dealing with an extremely large dataset offers a good chance for bioinformatics data processing. The ability and feasibility for underlying applications have been discussed [1,2].

We design a graph-based method for the NGS reads assembly problem and implement it as a software package, SeedsGraph. In the Background section, the NGS reads assembly problem and the framework for cloud computing are discussed. The Algorithm section presents the seeds definition and the related algorithms. The result of the experiments is presented in the Result section. Then, finally, there is a discussion about the assembly and results in Discussion and future work.

## Background

Genetic information of living organisms is stored in a chain of DNA molecules. There are four possible small molecules (also called nucleotides or bases): adenine (A), cytosine (C), guanine (G) and thymine (T). With the four-letter alphabet {A, T, G, C} we can represent the entire genetic information in strings. DNA molecules are denoted as a long string from the alphabet, duplicated and broken into fragments randomly for sequencing, which is also called shotgun sequencing. The whole genome shotgun (WGS) *de novo *assembly problem is the reconstruction of the genetic sequence information from a set of reads sequenced from the fragments. The shotgun process takes reads from random positions along a target molecule [3]. The WGS *de novo *assembly refers to the reconstruction in its pure form, without consultation to previously resolved sequence. For NGS data, this is a specialized problem due to the short length of reads and the large volumes of NGS data.

Sanger sequencing or conventional sequencing has been fine tuned to achieve read lengths of up to 1,000 base pair and per-base accuracies as high as 99.999%, but the amount of data produced is relatively small in each experiment and costs are great. However, NGS achieves much higher throughput with dramatically lower cost, because of the much higher degree of parallelism and much smaller reaction volumes. NGS has rapidly become prevalent since 2005 in research laboratories among companies and institutes. More and more NGS data are therefore produced and accumulated, and the demand for tools and methods for processing these data are increasing. But NGS has several fundamental limitations, especially for the assembly problem. The read length is remarkably short compared with Sanger sequencing and the number is huge. For example, the HiSeq2000 sequencer (Illumina) produces billions of 100 base pair reads with a total length of up to 600 gigabase pairs [4]. The error rates are relatively higher, and have a different distribution among the NGS technologies. The WGS overcomes these limitations by oversampling the target genome with short reads from random positions. Assembly software reconstructs the target sequences.

### Current assembly methods

The classical approaches for WGS *de novo *assembly have three steps: overlap, layout and consensus (OLC). In the first overlap step, the assembler computes all suffix-prefix alignments between each pair of reads, and builds a corresponding overlap graph. In the second layout step, the reads are nailed to the proper position based on the graph. In the last consensus step, each position in the target sequence is determined by several related reads. The OLC approach was typical in the Sanger data assemblers and was optimized for large genomes. There are many typical OLC software programs, such as Newbler [5], Celera Assembler [6], Arachne [7] and so forth. Besides these, there are string graph-based assemblers such as SGA [8] and Readjoiner [9], which take advantage of the FM**-**index and are derived from the compressed Burrows-Wheeler transform. The bottleneck of OLC methods is the computation of pairwise suffix-prefix alignment for the overlap graph, and it is the most time-consuming and space-consuming task.

An alternative approach is widely applied to the NGS short reads based on the de Bruijn graph, a kind of *k*-mer graph whose attributes make it attractive for vast quantities of short reads. The de Bruijn graph does not require the computation for all pairs of reads, but enumerates all *k*-mers for every read and joins them into a path. The graph itself does not store individual reads or their overlaps, and compresses redundant sequences. There are also many typical de Bruijn graph-based software programs, such as Velvet [10], EULER-SR [11], Abyss [12], and so forth. However, reducing short reads into even shorter units compromises the ability of disambiguation of short repeats. Another disadvantage is the loss of long-range continuity information in reads.

In this paper, we present a heuristic graph-based greedy algorithm for the assembly of NGS short reads. We utilize the MapReduce framework for computation of intensive short reads overlapping work, and then cluster them into groups. The clusters are compressed into chains of seeds, and then a seeds graph is built from chains by seed overlapping. Finally, contigs are threading from the seeds graph after optimization for repeats and sequencing errors. A main advantage of building graphs by chains is that each chain in a cluster represents a valid assembly of reads. We provide the SeedsGraph package, which using the open-source implementation of a distributed programming framework MapReduce [13] called Hadoop [14]. The result shows that SeedsGraph is efficient and feasible for NGS data. Furthermore, SeedsGraph can use remote computing services over the Internet such as the Amazon Elastic Compute Cloud [15].

### MapReduce and Hadoop

MapReduce is a software framework designed and used by Google to support parallel distributed processes of large data-intensive applications and services [13]. Google uses this framework internally for their services such as Gmail, Docs, and so forth, and processes petabytes of data all on commodity computers. This framework uses two phases called map and reduce, which borrow from functional programming to split data into small pieces for processing and then merging the results. Unlike common parallel computing frameworks, MapReduce does not need a developer to explicitly manage data distribution, interprocess communication or network access. The framework automatically executes these functions (maps and reduces) by a job controller in parallel over any number of processors utilizing both multiprocessors in one node and multinodes in computers of a network cluster. Between the map and reduce phases, there is also a shuffle phase for shuffling and sorting intermediate data distributed over nodes in the network. The shuffle phase could also be used as a temporary processing or filtering step for intermediate results in the middle of a whole job.

**Figure 1:**
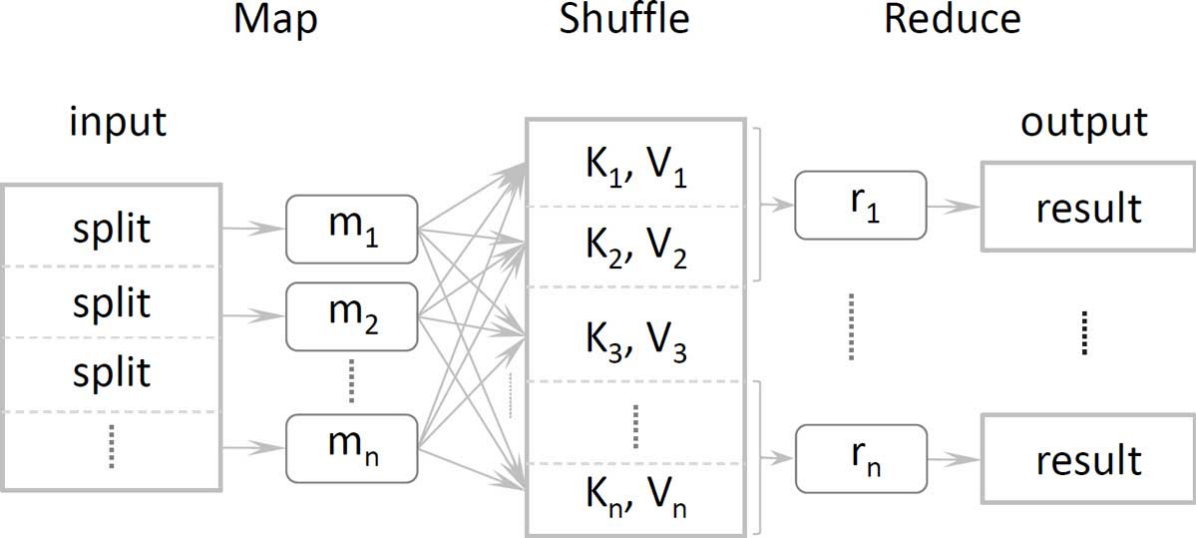
**MapReduce framework**.

As shown in Figure 1, the map function generates key-value tuples from the input data. One record of input data could be related to none, one or multiple key-value tuples based on the difference of applications. For example, for the word count problem, to compute all occurrences of a number of words, the map function could emit all (word, 1) tuples for each word from a single line of an input document. For larger input data, the framework could run the map function in parallel on multiprocessors. Once the mappers are complete, the result tuples are shuffled by MapReduce and grouped by the keys. The result is a large distributed hash table indexed by the keys, and each key is related to a list of values. For the word count problem, every world has a list of 1s as values. The reduce function could then be any function with input of a single key and a corresponding list of values. So for the word count problem, the reduce function could just sum up all 1s into a final count result. In general, the reduce function should be commutative, since the order of the tuples is unstable and distributed across the network. The MapReduce framework ensures all tuples with the same key are transferred to same computer node and executed by the same reduce function. Each instance of the reduce function therefore executes only dependent tuple keys from the map function, and there can be as many reduce functions executing in parallel as the number of keys.

In other distributed computing frameworks, the architecture is more computing intensive rather than data that are stored in some specific data nodes. The computing nodes obtain necessary data from nodes through a high-bandwidth network. Computing nodes and data nodes are separated by network. However, MapReduce is designed for an extremely large dataset, far beyond the RAM size, even local disks, and it is not feasible for frequent network IO. Google designed a new specialized distributed Google File System (GFS) to efficiently support MapReduce jobs [16]. In comparison with other distributed systems, every node in MapReduce is both for computing and for data storage. The MapReduce framework then actually moves the computing to the data node on commodity computers; that is, the cluster. GFS is designed to provide very high-bandwidth data IO for MapReduce by replicating and partitioning files across many physical disks. When data are transferred into GFS, they are copied several times on nodes across the network, and when MapReduce starts a job, it will use local data first instead of fetching remote data.

Hadoop and the Hadoop Distributed File System (HDFS) are excellent open-source implementations of Google's MapReduce and GFS respectively in Java and are sponsored by Amazon, Yahoo, Google, IBM, and so forth. Application developers in Hadoop need only write custom map and reduce functions in Java or any other language using Hadoop Streaming technology. The framework will automatically execute them in parallel. Hadoop and HDFS are capable of managing clusters with thousands of nodes and petabytes of data. There are benchmarks showing that Hadoop is capable of a very large amount of efficient data processing.

*Algorithm 1. MapReduce *k*-mer Group Algorithm*

**Input: ***R*, the set of all reads

**Output: ***K*, the set of *k*-mer Group

**1:procedure **MAP(*seq, seq id*)

**2:  for all ***k*-mer *m_k _***in ***seq *do *// *∀*seq *∈ *R*

**3:    **Emit a tuple (*m_k_, OffsetInfo*)

4:  end for

5:end procedure

**6:procedure **REDUCE(*m_k_, N*) *// N*: *OffsetInfo *set

**7:  if **1 *<*CountSeqs(*N*) ≤ *t ***then**

**8:    **Emit a *k*-mer group (*m_k_,N*) into *K*

**9:  end if ***// t*: the max number of reads sharing a *k*-mer

10:end procedure

### Algorithm

In the consideration of a very large number of NGS reads, we cluster reads according to seeds, which is an approximate substring shared by reads with some extra information and will be defined properly in Definition 2. But raw reads clustering is also a space-consuming and time-consuming task, so we use the MapReduce framework on it. A read cluster is denoted by a chain of related seeds, which have all records about the reads in it. Then a seeds graph (Definition 4) is built based on the seeds' overlapping relationship and paired-end information, and the graph is used to direct the assembly.

### Generating the *k*-mer group

**Figure 2:**
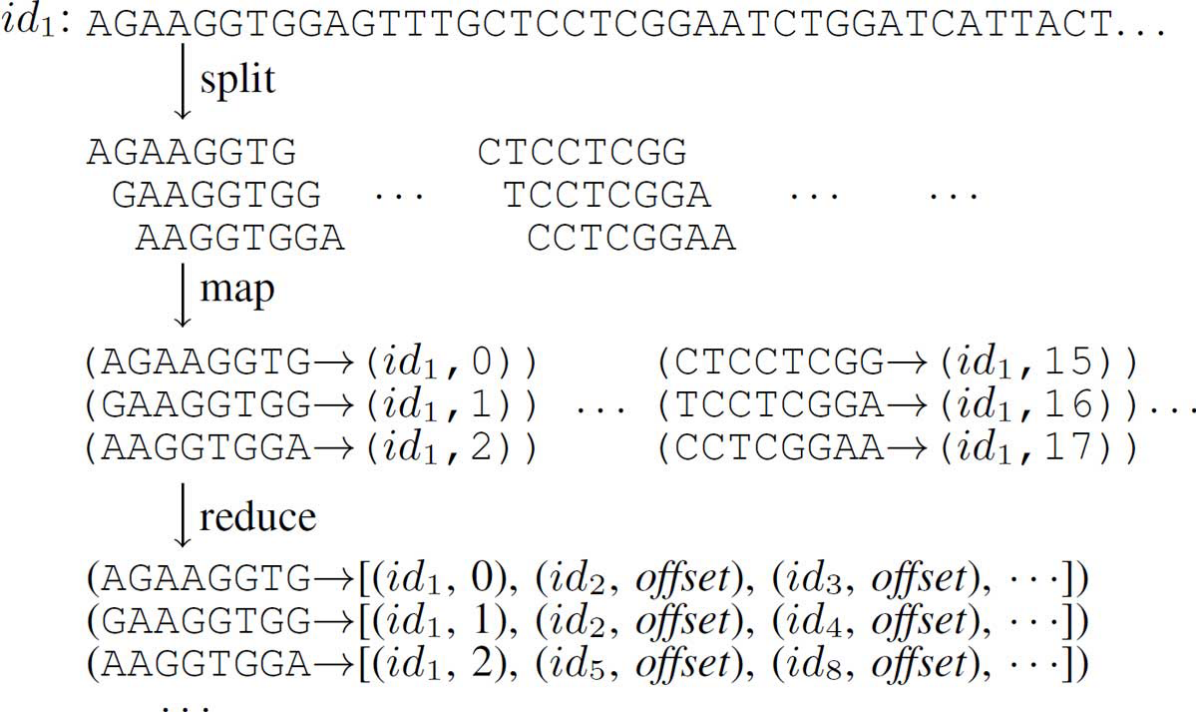
**Generating the *k*-mer Group from read sequences with MapReduce**.

We employ the MapReduce framework for the generation of *k*-mer groups used in read clustering, as detailed in Algorithm 1. The map function of MapReduce scans each part of the input sequence (that is, raw read) in *R*, which is the set of all reads, and emits key-value pairs in the form of (*k-mer, OffsetInfo*), where *OffsetInfo *is a tuple of (*seq id, offset in seq*). The *seq id *is the sequence's unique ID from the mapper's input, and the *offset in seq *is the position of the *k*-mer inside the sequence. Through an iteration over the sequence, mappers can collect all length-*k *substrings (*k*-mers) and offsets (*offset in seq*s) (see Figure 2). The sequence is then converted into its reverse-complement form for all reverse-complement *k*-mers. To distinguish from original *k*-mers, *offset in seq *of reverse complements is marked as a negative integer value. By a single run of a map function, we can obtain all *k*-mers and reverse-complement *k*-mers of a sequence. Since there is no relation between each two of reads in this step, the execution over all reads could be parallelized without any loss of accuracy. Once all mappers are completed, Hadoop would shuffle the key-value pairs, and group all values with same *k*-mer key into a long list, the *k*-mer Group, in the reduce function.

**• Definition 1 (k-mer Group): **A *k-*mer Group is a set of tuple (*m_k_,N*), where *m_k _*is a *k*-mer and *N *is a set of *OffsetInfo*, which means all sequences in *N *sharing *m_k _*in a specified position.

In genome data, there are many highly repeated areas, which are full of the same or similar sequences. If a *k*-mer is one inside these regions, its huge *k*-mer group is hazardous for assembly. We therefore limit the *k*-mer group size with a threshold *t*, and the reduce function will drop it automatically. The output of Algorithm 1 is a complete valid *k*-mer group set, denoted as *K*. The complexity of a map job for *k*-mer generating is in *O*(*nm*) time and *O*(4*^k^m*) space, where *n *is the number of reads and *m *is the length of a read.

### Reads clustering

Reads clustering is the core part in this assembly package. We use clustering to eliminate the large amount of NGS reads, split reads into clusters, and denote the cluster as a series of short substrings; that is, seeds. The strategy about reads clustering detailed in Algorithm 2 is as follows: take a sequence from reads randomly as a cluster center; find all sequences sharing a *k*-mer with the center; and validate each sequence by extend *k*-mer to *seeds*.

A cluster center *r_c _*is randomly taken in line 3, and the related sequences denoted by set *T *are gathered from *K *by enumerating all *k*-mers of *r_c _*in lines 5 to 8. Firstly, we compare all reads related to all *k*-mers with *r_c_*. If a read shares a block with *r_c _*with enough length, we could just align the read with *r_c_*. The existence of enough length could be concluded from the *k*-mer uniqueness discussed in [17]. Then, other reads are aligned to *r_c _*by those sharing *k*-mer and positions.

**Figure 3:**
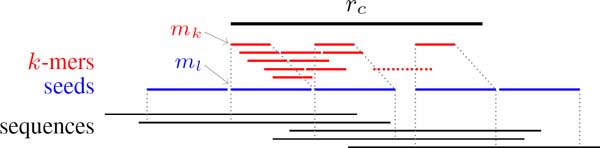
**Extension from *k*-mer *m_k _*to *l*-mer *m_l _*and seeds building in a cluster (the seeds outside *r_c _*are built from sequences aligned to *r_c_*)**.

In the next step, we extend a *k*-mer to an *l*-mer in *r_c_*, as illustrated in Figure 3, where *k <l *holds, and filter out all sequences that there are more than *e *mismatch in *l*-mer compared with *r_c_*. A seed is created from the *l*-mer and related sequences including *r_c _*information. The length of *l *relies on the uniqueness of substrings of the genome, which was discussed comprehensively in [18] especially for large repetitive plant genomes. For any *l *where 4*^l ^*exceeds twice the genome size, most *l*-mers should be unique [19]. We choose *l *as a slightly smaller value for the uniqueness and error corrections.

**• Definition 2 (seed): **A *seed *is a 4 tuple (*id, m_l_, seq list, next*). The *id *is a unique number for all seeds, the *m_l _*is a *l*-mer which is shared by the sequences list in *seq list *which is copied from the *k*-mer group. The *next *is a field for the following seeds or *none*, and stores the next seed's *id *and the distance which could be a negative value if overlapping with the following one. If sequences have mismatch less than *e*, then mismatch positions is denoted in *m_l _*as 'n' instead of the original 'A', 'T', 'C' or 'G'.

For all *k*-mers in *r_c_*, one will check all sequences sharing *k*-mer, but only nonoverlapping seeds will be emitted, as detailed in lines 11 to 22. For alignment outside *r_c_*, we use the same strategy emitting nonoverlapping seeds from other sequences next to both ends of *r_c_*, as in lines 23 to 26. Because the output seeds are ordered along the alignment in line 7, we join the seeds in a *chain *by the *next *field.

**• Definition 3 (seeds chain): **A *seeds chain *or simply *chain *is a linked list of seeds which is a representation of a cluster centered on *r_c _*and the alignment of related sequences.

Whereas the seeds in a chain cannot have any overlapping in one cluster, the distance between each two consecutive seeds could be spaced, because of some nonexisting *k*-mers filtered out by the reduce function in Algorithm 1. When all seeds covering the alignment in line 7 are generated, we finish a candidate cluster - which is all sequences in the seeds. In the last step, if a sequence only shares a single seed with others, it is not suitable for this cluster. So we define a ration threshold *h*, which is the coverage of a sequence by the cluster chain, as in lines 27 to 32. If a sequence's coverage is under *h*, it is removed from the cluster. Finally, the cluster center on *r_c _*is complete, and has been denoted by a chain *S *that is saved into the result chain set *Q*. We update *R *by removing the sequences of *S*, then iterate it over *R *until empty to finish the algorithm.

The clustering algorithm runs on Hadoop for HDFS only; the jobs are controlled by a separate job controller instead of MapReduce. Each cluster is gathered by one task in a node of the Hadoop environment, but random selection for the cluster center should be distinguished among different nodes. So we use a self-defined job controller to select the center sequence, then send it to a node in Hadoop for a clustering task, as in line 3. The task firstly gathers the temporary set *T *of necessary sequences from *R *on HDFS, then saves it on HDFS in line 9. The job controller monitors HDFS for *T*, and then selects another cluster center from *R *− *T *for another job. So the reads clustering algorithm runs parallel in the Hadoop environment with the help of HDFS.

For a given read *r_c_*, there are total (*m *− *k*) *k*-mers and *n_rc _*related reads. The alignment of each read to *r_c _*by sharing the *k*-mer is in *O*(*n_rc_*) time, but the *l*-mer extension needs *O*(*l*) and *O*(*m*) for the worst case. For the job of seeds construction and cover ration, *O*(*n_rc_*) is calculated. So the total is in *O*(*ln_rc_*), and for the worst case is *O*(*mn_rc_*).

### Seeds graph building

**• Definition 4 (seeds graph): **A *seeds graph G *is a tuple (*V,E*), where *V *is a vertex set and *E *is a edge set. The vertexes are identical with seeds generated by clustering algorithm. The edges are the union from edges in each of chain in *Q *and the suffix-prefix overlapping relation calculated from all pairs of seeds that do not belong in the same chain.

A seeds graph is essentially a *l*-mers overlapping graph, where *l*-mers are from seeds generated by a clustering algorithm, but the two vertexes of overlapping edges are only from different clusters. The chain set *Q *is already in a seeds graph structure with vertexes and chain edges, and we only need to calculate all overlapping edges and update *Q *with connected components. Short cluster contigs will therefore be joined together across tail seeds at both ends inside a cluster.

The *l*-mers in all seeds have duplications with errors because of different clusters, so we firstly merge all seeds with the same pattern for less suffix-prefix overlapping computation. If seeds are merged into a single seed successfully, we use the smallest seed ID as the new seed and update all error positions. This is done in Algorithm 3 lines 2 to 7. The seeds merge algorithm is trivial, but the error correction should be considered. One simply aligns reads according to the *l*-mer and merges reads into a single one, then recalculates the mismatch position among sequences inside the *l*-mer window. The merge process is also called a spectral alignment in other assemblers such as Euler [20].

In lines 8 to 13, we initialize the graph *G *by adding all vertexes and edges in *Q*. Then all overlapping edges are checked by computing the suffix-prefix alignment among all pairs of seeds in lines 14 to 18. The running time for calculation of seeds overlapping in Algorithm 3 is *O*(*l*^2^). But we need to compare all pairs of seeds, so the total running time is *O*(*n*^2^*_s_l*^2^), where *n_s _*is the number of seeds.

### Contigs and repeats

**Figure 4:**
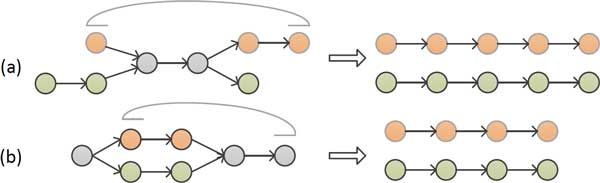
**Methods to resolve graph complexity**. (a) Split sharing in-path nodes by a mate-pair (b) Split sharing starting and ending nodes by a mate-pair

Using seeds instead of reads, the seeds graph construction discards long-range continuity information in reads. We repair this by threading the reads through the graph with the help of pair-end information. Actually the reads in a cluster center have already been done. Paired ends that span a repeat provide the evidence to join one chain that enters a repeat to another chain that exits the repeat (Figure 4a). A seeds graph may have several paths between nodes related to two ends of a mate pair. One path implies a putative contig, and only one of the paths implies a sequence whose length satisfies the pair-end constraint as in Figure 4b.

It is not possible to simplify all parts of the graph. So if a repeat is shorter than the cluster length or the mate-pair insert size, it is simplified as much as possible; and if not, it would be kept as original. In general, branching and convergence increases graph complexity, leading to tangles that are difficult to resolve. If a contig has been identified, all related reads will be removed from the graph. The remaining connected components would be smaller while the iterations run over the graph. Finally, contigs produced from analysis in the graph are in mate pairs for scaffolds such as in classic OLC assemblers.

*Algorithm 2. Reads Clustering Algorithm*

**Input: ***R *and *K*, the read set and the *k*-mer group set

**Output: ***Q*, the set of chains for read clusters

**1:procedure **READSCLUSTER(*R, K*)

**2: while ***R *≠ ∅ **do**

**3:  **Take a *r_c _*∈ *R *randomly by job control

**4: **  *T *←∅; *C *←{*r_c_*}

**5:  for all ***k*-mer *m_k _***in ***r_c _***do**

**6: **    Merge sequences in *m_k _*group from *K *into *T*

**7: **    Align *T *to *r_c _*according to shared *k*-mers

8:  end for

**9: **  Save *T *to *HDFS *for next job

**10: **  *S *←∅; *d *← *none*

**11:  for all ***k*-mer *m_k _***in ***r_c _***do**

**12: **    Extend *m_k _*to a longer *l*-mer *m_l _*in *r_c_*

**13:    for all ***r *∈ *T *− *C ***do**

**14:      if **Mismatch(*r, m_l_*) *< e ***then**

**15: **        *C *← *C *∪{*r*}

16:      end if

**17:      if ***m_l _***and ***d *is **not **overlapping **then**

**18: **        *d*← Create a new seed from

**19: **        *_S _*←*_S _*∪{*_d_*}

20:      end if

21:    end for

22:  end for

**23:  for all ***m_l _*next to *r_c _***in ***r *∈ *C*(*r *≠ *r_c_*) **do**

**24:    ***d *← Create a new seed from *m_l_*

**25:    ***S *← *S *∪{*d*}

26:  end for

**27:  for all **sequence *r ***in ***C ***do**

**28:    if **CoverRatio(*r, S*) *< h ***then**

**29:      ***C *← *C *−{*r*}

**30:      **Remove *r *from seeds in *S*

31:    end if

32:  end for

33: end while

34:end procedure

## Result

The presented methods for constructing the seeds graph and the subsequent computation of contigs have been implemented in a sequence assembler named SeedsGraph, which provides versions both on Hadoop clusters and on a single host.

For our benchmark, we use the single host version for comparisons. The experiments' platform is a 64-bit Debian Linux computer with Intel Xeon E5-2620 CPU (15M cache, 2.00 GHz, six cores and 12 threads) and 64 GB RAM. We run SeedsGraph with maximum 24 threads at the same time if necessary. For SeedsGraph, the parameters *K *= 11 and *L *= 20 are used.

*Algorithm 3. Seeds Graph Building Algorithm*

**Input: ***Q*, the chain set for read clusters

**Output: ***G*, the seeds graph

**1:procedure **SEEDSGRAPH(*Q*)

**2: **Sort all seeds lexicographically of each chain in *Q*

**3: **Merge seeds with the same pattern

**4:  for all **chain *a ***and ***b *share consecutive seeds **do**

**5: **    *d *← Create a new chain from *a *and *b*

**6: **    Replace *a,b *with *d *in *Q*

7:  end for

**8: **  *V *← all seeds of each chain in *Q*; *E *←∅

**9:  for all **chain *c ***in ***Q ***do**

**10:    for all **each two consecutive seeds *u,v ***in ***c ***do**

**11:      **Add an edge (*u,v*) into *E*

12:    end for

13:  end for

**14:  for all **seeds *u,v *in *V *but not in one chain **do**

**15:    if **Overlap(*u*,*v*) *> q ***then**

**16:      **Add an edge (*u,v*) into *E*

17:    end if

18:  end for

**19: **Output *G *= (*V,E*)

20:end procedure

We chose WGS data from four deep-coverage sequencing projects, and took the dataset of *Staphylococcus aureus *and *Rhodobacter sphaeroides *from [17]. Data for *S. aureus *and *R. sphaeroides *could be downloaded from the Sequence Read Archive at NCBI, accession numbers SRX007714 and SRX016063 [21]. The data are detailed in Table 1.

**Table 1:** Details of next-generation sequencing datasets used for experiments

Species	*Staphylococcus aureus*	*Rhodobacter sphaeroides*
Size (Mb)	2.9	4.6
Read length	101	101
Insert size (base pairs)	180	180
Number of reads	1,294,101	2,050,868

We selected another assembler, SGA [8], as a comparison. All methods for experiments refer to [17], but the parameters may be different from the same dataset. We run SeedsGraph on a single host instead of on a cluster for the comparisons. The resulting experimental data are detailed in Table 2.

**Table 2:** Assembly result of next-generation sequencing data for *Staphylococcus aureus *and *Rhodobacter sphaeroides*

Dataset	*Staphylococcus aureus*		*Rhodobacter sphaeroides*	
	
	**SGA **[8]	SeedsGraph	**SGA **[8]	SeedsGraph
Number of contigs	274	754	3,067	7,033
N50 of contigs (kb)	24	43	27	42
Number of scaffolds	122	323	2096	4291
N50 of scaffolds (kb)	205	174	95	46
Assembly size (%)	94.2	87.4	97.2	86.3

The parameter settings are a dilemma about longer N50 and good coverage in SeedsGraph. Contig or scaffold N50 is a weighted median statistic such that 50% of the entire assembly is contained in contigs or scaffolds equal to or larger than this value. If we use a more stringent seeds setting, the resulting clustering will be smaller and the N50 will be shorter; and otherwise there will be lots of singleton clusters (only one read in it) that would be discarded.

## Discussion and future work

In this paper we present methods and implementation techniques for a new clustering-based, graph-conducted assembler, named SeedsGraph, which is efficient and takes advantage of cloud computing for the large dataset of NGS data.

In the software package, we provide tools both for the Hadoop version and for the single host version assembler. Although the different graph-based assemblers aim at constructing the overlapping graph, they apply different heuristics to compute a layout from the graph; we also use the idea of the de Bruijn graph in the seeds graph. Our main development is about the new clustering algorithm based on *k*-mer sharing in both the MapReduce framework and the single host platform. The necessary techniques we use are related in basic computer theory about sequence processing, such as the longest common substring and so forth. The seeds graph is inspired from the de Bruijn graph in assembly short reads. We provide the software package online [22]. The Java source code for the Hadoop version and the python source code for the single host version are both available. Any comments and suggestions are welcome, and readers should feel free to contact the authors.

In the future, we will expand the performance and utility spectrum of SeedsGraph on several levels. First, we will optimize the method by further improving its running time and ability for larger genomes. Second, we will improve the seeds structure for better fuzzy string matching for better clustering results. Finally, additional input and output formats will be implemented in SeedsGraph to provide support for a wide spectrum of upstream and downstream software tools and programming environments.

## Abbreviations

GFS, Google File System; HDFS, Hadoop Distributed File System; NGS, next-generation sequencing; OLC, overlap, layout and consensus; WGS, whole genome shotgun.

## Competing interests

The authors declare that they have no competing interests.

## Authors' contributions

All authors had made substantial contributions to conception and design, or analysis and interpretation of data. CW detailed the algorithms and programs, and drafted the manuscript. MG and QZ contributed to project design and involved in drafting the manuscript. XL and YL assisted in the analysis and data acquisition. All authors approved the final manuscript.
